# Macrophage subsets exhibit distinct *E*. *coli*-LPS tolerisable cytokines associated with the negative regulators, IRAK-M and Tollip

**DOI:** 10.1371/journal.pone.0214681

**Published:** 2019-05-23

**Authors:** Khalid Al-Shaghdali, Barbara Durante, Christopher Hayward, Jane Beal, Andrew Foey

**Affiliations:** 1 School of Biomedical Sciences, Faculty of Medicine & Dentistry, University of Plymouth, Drake Circus, Plymouth, United Kingdom; 2 College of Medicine, University of Hail, Hail, Kingdom of Saudi Arabia; 3 Department of Gastroenterology, Derriford Hospital, Plymouth, United Kingdom; 4 School of Biological and Marine Sciences, University of Plymouth, Drake Circus, Plymouth, United Kingdom; Medical Center - University of Freiburg, GERMANY

## Abstract

Macrophages (Mϕs) play a central role in mucosal immunity by pathogen sensing and instruction of adaptive immune responses. Prior challenge to endotoxin can render Mφs refractory to secondary exposure, suppressing the inflammatory response. Previous studies demonstrated a differential subset-specific sensitivity to endotoxin tolerance (ET), mediated by LPS from the oral pathogen, *Porphyromonas gingivalis* (PG). The aim of this study was to investigate ET mechanisms associated with Mφ subsets responding to entropathogenic *E*. *coli* K12-LPS. M1- and M2-like Mφs were generated *in vitro* from the THP-1 cell line by differentiation with PMA and Vitamin D_3_, respectively. This study investigated ET mechanisms induced in M1 and M2 Mφ subsets, by measuring modulation of expression by RT-PCR, secretion of cytokines by sandwich ELISA, LPS receptor, TLR4, as well as endogenous TLR inhibitors, IRAK-M and Tollip by Western blotting. In contrast to PG-LPS tolerisation, *E*. *coli* K12-LPS induced ET failed to exhibit a subset-specific response with respect to the pro-inflammatory cytokine, TNFα, whereas exhibited a differential response for IL-10 and IL-6. TNFα expression and secretion was significantly suppressed in both M1- and M2-like Mφs. IL-10 and IL-6, on the other hand, were suppressed in M1s and refractory to suppression in M2s. ET suppressed TLR4 mRNA, but not TLR4 protein, yet induced differential augmentation of the negative regulatory molecules, Tollip in M1 and IRAK-M in M2 Mφs. In conclusion, *E*. *coli* K12-LPS differentially tolerises Mφ subsets at the level of anti-inflammatory cytokines, associated with a subset-specific divergence in negative regulators and independent of TLR4 down-regulation.

## Introduction

Endotoxin tolerance (ET) is a phenomenon where cells become hypo-responsive to endotoxin/lipopolysaccharide (LPS), unable to respond to repeated LPS challenge. ET has been studied widely *in vivo* and *in vitro* in both animals and humans [1]. ET can be both beneficial and detrimental to both the host and pathogen alike; immune suppression in general, will benefit the host by dampening down harmful host-derived inflammatory responses that result in tissue degradation, whereas this suppression will also give the pathogen a reprieve from hostilities, enabling expansion of pathogen numbers. Tolerance induction and sensitivity to ET is fundamental to the homeostatic function of the gut mucosa; effectively allowing the gastrointestinal tract to determine immune fate, tolerating safe non-self, such as commensal microbes and food whereas maintaining the ability to be activated in inflammatory responses mounted to unsafe non-self, pathogenic material. Breakdown or dysregulation of tolerance is fundamental to gut pathology such as inflammatory bowel disease (IBD) or colorectal cancer (CRC). Gut mucosal macrophages (Mφs) are essential to ET; their differentiation and activation status is indicative of whether the mucosal environment is harmful to the pathogen or host tissue. ET induced in Mφs results as a consequence of many different mechanisms, which include induction and responsiveness to anti-inflammatory cytokines (e.g. IL-10 and TGFβ), down-regulation of PRRs (e.g. TLR4), shedding of cytokine receptors and PRRs and induction of negative regulatory molecules, which have a functional role in inhibition of TLR4 signal transduction, such as Tollip, Myd88s, SARM, IRAK-M and SIGIRR (reviewed in [1]).

Mucosal Mφs have a dual functionality that determines tolerance to commensal organisms or immune responsiveness to entropathogens such as *E*. *coli*. This homeostatic tolerisation phenotype is associated with the M2 Mφ subset whereas an immune activatory/pro-inflammatory phenotype is associated with the M1 Mφ subset (reviewed in [2]). The immune-suppressive anti-inflammatory function of M2 Mφs resembles features of ET; exhibiting a predominant induction of anti-inflammatory cytokines (e.g.IL-10) and a corresponding down-regulation of pro-inflammatory cytokine induction (e.g. TNFα) [3]. It is an oversimplification however, to assign the phenomenon of ET to a specific Mφ subset due to the ever-increasing characterisation of many varieties of Mφ subsets according to their differentiation, stimulation, pathological and tolerisation status [4]. Previous investigations in this laboratory have suggested both differential and overlapping sensitivity to suppression between M1 and M2 Mφ subsets, determined by phenotype of subset, PAMP and corresponding recognition by PRR [5]. In general, LPS-tolerisation of downstream Mφ immune responses is determined by both membrane-associated and intracellular signaling mechanisms; whereby membrane-associated mechanisms include reducing expression of TLR4 surface protein, PAMP-binding and sensitivity of Toll like receptors, and absence of required co-receptor subunits MD2 and CD14 [6–8]; such mechanisms are linked to the homeostatic functionality of M2-like mucosal Mφs in healthy gut mucosa. With respect to intracellular signalling mechanisms, endogenous suppressors such as Tollip, IRAK-M and short version of MyD88 (MyD88s) have been linked to TLR4-mediated LPS unresponsiveness [1].

*Escherichia coli* LPS has been shown to differ from *P*. *gingivalis* LPS in structure and various functional activities [9,10], where *E*.*coli* K12 LPS generally exhibits a more robust endotoxin activity than that displayed by PG-LPS [5]. Indeed, previous investigation demonstrated that pre-treatment of THP-1 monocytic cells with *E*. *coli* and PG-LPS differentially modulate cytokine production and CD14, TLR2, and TLR4 surface expression. This study reported a significant difference in the relative potencies of *E*. *coli* LPS and PG-LPS to induce pro-inflammatory cytokines, where PG*-*LPS was a less effective inducer of pro-inflammatory cytokines. This differential response could also be observed with the induction of ET. Pre-treating THP-1 cells with 100 ng/ml *E*. *coli* LPS resulted in 90% reduction in TNFα, IL-1β and IL-6 production with no effect on CD14 expression, whereas pre-treatment with the same concentration of PG-LPS resulted in a significant decrease of IL-1β and enhancement of surface CD14 expression [10]. A similar study, again using THP-1 monocytic cells, showed that ET induced by *E*. *coli* LPS and PG-LPS suppressed TNFα, IL-1β and increased IL-10, whereas ET induced by *E*. *coli* alone resulted in a suppression of IL-8. Down-regulation of TLR2 or TLR4 protein was observed in cells tolerised with PG-LPS or *E*.*coli* LPS, respectively. After retreatment with PG-LPS or *E*. *coli* LPS, the expression levels of the intracellular negative regulators of TLR signalling, IRAK-M was increased, whereas Tollip was unaffected [11].

When considering potential differences in M1 and M2 Mφ subset responses to endotoxin, PG-LPS exhibited a lower endotoxin activity than *E*.*coli*-LPS with respect to secretion of inflammatory cytokines and chemokines produced from polarised murine bone marrow-derived Mφs [12]. Previously, endotoxin tolerisation studies have described a differential suppression between M1 (pro-inflammatory, CD14^hi^) and M2 (regulatory, CD14^lo^) Mφ subsets in response to LPS of an oral pathogen, *Porphyromonas gingivalis*. This differential sensitivity of Mφ subsets to ET described the pro-inflammatory M1-like subset to be refractory to tolerance induced by *P*. *gingivalis* LPS (little or no suppression of TNFα, IL-1β, IL-6 and NFκB activity), whereas the M2-like subset was sensitive to tolerance induced by *P*. *gingivalis* LPS and suppressed inflammatory cytokines TNFα, IL-1β, IL-6 and NFκB activity [5]. In addition, this differential Mφ subset, sensitivity to ET has been suggested to be further reinforced by the differential induction and responsiveness to the anti-inflammatory cytokine, IL-10 [13]. *E*. *coli*, on the other hand, is an intracellular gut mucosal pathogen; *E*. *coli*-LPS is understood to be able to induce ET in macrophages [10,11,14,15]. The aim of this study was to investigate the susceptibility of these distinct M1 and M2 Mφ subsets to *E*. *coli* K12-LPS-tolerance and to characterise the mechanisms that underpin this tolerance induction.

## Materials and methods

### Macrophage (Mϕ) culture

THP-1 cells were maintained in RPMI-1640 medium supplemented with 10% v\v foetal calf serum (FCS), 2mM L-glutamine and 100 U/ml Penicillin and 100 μg/ml Streptomycin (Lonza, Wokingham, UK) (here on referred to as R10) at 37^o^ C, 5% CO_2_ incubation. THP-1 cells were differentiated into M1-like Mφ subset (Pro-inflammatory) and M2-like subset (anti-inflammatory) by incubation of THP-1 monocytic cells in the presence of 25ng/ml phorbol-12-myristate acetate, PMA (Sigma-Aldrich, Poole, UK) for 3 days and 10nM 1,25-(OH)_2_ -Vitamin D_3_ (Sigma-Aldrich, Poole, UK) for 7 days for M1-like and M2-like Mφ subsets, respectively. Prior to experimentation, M1-like Mφs were washed and incubated for an additional 24 hours to washout PMA. This washout protocol ensured low background cytokine production and that cytokine responses measured were directly induced by *E*.*coli* K12-LPS and not by the residual activation by the diacylglycerol (DAG) analogue, PMA, used to differentiate THP-1 cells to Mφs.

PMA- and Vitamin D_3_-differentiated THP-1 cells were used as an appropriate model of primary blood monocyte-derived M1 and M2 Mφs polarised by GM-CSF or IFNγ and M-CSF or IL-4/IL-13, respectively. These cell line-derived Mφ subsets exhibited a similar phenotype to the primary monocyte-derived M1 and M2 Mφ subsets, where the PMA (M1-like) MФs were TNFα^hi^ (mRNA & protein), IL-12^hi^, IL-10^lo^ (mRNA), endogenous IL-10 activity^-^, IL-6^hi^, iNOS^+^ (mRNA), Arginase^-^ (mRNA), Dectin-1^-^, CD206^-^, phagocytic activity^+^ and the Vitamin D_3_ (M2-like) Mφs were TNFα^lo^ (mRNA & protein), IL-12^lo^, IL-10^hi^ (mRNA), endogenous IL-10 activity^+^, IL-6^lo^, iNOS^-^ (mRNA), Arginase^+^ (mRNA), Dectin-1^+^, CD206^+^, phagocytic activity^++^ [5,11,13, 16–18].

### Activation of macrophage (Mφ) subsets

THP-1-derived M1- and M2-like Mφs were stimulated by 100 ng/ml *E*.*coli* K12-LPS (Invivogen, Toulouse, France) for defined time periods at 37^o^ C/5% CO_2._ Afterwhich time, the conditioned supernatants were harvested and stored at -20^o^ C until required for cytokine assay by sandwich ELISA, whereas cell lysates were used for detection of gene expression by Real Time polymerase chain reaction (RT-PCR) and intracellular proteins by Western blotting.

### Tolerisation by pre-incubation with *E*.*coli* K12-LPS

THP-1-derived M1- and M2-like Mφs were pre-treated with 100 ng/ml K12-LPS for 4 hours and 24 hours, afterwhich time the pre-stimulus culture medium was removed carefully and Mφs were washed in fresh R10 before re-stimulation by 100 ng/ml K12-LPS for a further 18 hours at 37^o^ C/5% CO_2_. The conditioned supernatants were harvested and stored at -20 ^0^C until required for cytokine assay by sandwich ELISA whereas cell lysates were used for detection of gene expression by Real Time polymerase chain reaction (RT-PCR) and intracellular proteins by Western blotting. To demonstrate a physiologically-relevant tolerisation; after stimulation or tolerisation protocols, Mφ viability was routinely checked by Trypan blue (Sigma-Aldrich, Poole, UK) exclusion. No significant reductions in Mφ viability were observed for stimulation/tolerisation protocols used in this study, viability was routinely >85%.

### Cytokine measurement

At the end of the culture period, conditioned supernatants were harvested and stored at -20 ^0^C. The level of TNFα, IL-6 and IL-10 secretion into the culture supernatants was determined by sandwich ELISA using capture and detection antibodies commercially available from R&D Systems UK Ltd., Abingdon and BD Pharmingen, Oxford, UK. Protocols were followed according to manufacturer’s instructions and compared to standard curves between the range of 7 to 5,000 pg/ml, using the international standards available from NIBSC, Potter’s Bar, UK.

### Real-time PCR analysis of gene expression

Expression of TNFα, IL-6, IL-10, TLR4, IRAK-M, Tollip and glyceraldehyde-3-phosphate dehydrogenase (GAPDH) mRNA was evaluated by real-time polymerase chain reaction (RT-PCR). Following each treatment, cells were washed with ice-cold PBS and total RNA was isolated using GenElute RNA extraction kit (Sigma-Aldrich, Poole, UK) according to manufacturer’s instructions. The total RNA concentration was determined using NanoVue spectrophotometer (GE Healthcare, Freiberg, Germany). RNA purity was assessed by examining the absorbance ratio at 260 and 280 nm. One microgram of total RNA was reverse transcribed using MMLV Reverse Transcriptase reaction kit (Sigma-Aldrich, Poole, UK). Sequence specific primers for the target mRNAs (Table 1) were designed using Primer Express Software (Applied Biosystems, Paisley, UK) and synthesised by Eurofins MWG Operon (Ebersberg, Germany). RT-PCR was performed using StepOnePlus thermal cycler and Power SYBR Green kit (Applied Biosystems, Foster City, CA, USA) using 10 pmol of the forward and reverse primers for each target. Target amplification was carried out under the following conditions: preheating at 95°C for 10 min, followed by 40 cycles at 95°C for 30 s, 60°C for 1 min and 72°C for 1 min. RT-PCR data were analysed following the 2^-ΔΔCt^ method as described by Livak and Schmittgen [19], using GAPDH as an endogenous control and resting cells as a reference sample. Thus, the relative quantity of the target transcript is described as fold change (RQ, relative quantitation) relative to the reference sample and GAPDH.

**Table 1:** Sequence of real-time PCR primers and estimated product size. Oligonucleotide sequences are presented for the forward and reverse primers for the cytokines, TNFα, IL-6 and IL-10, the negative regulatory molecules Tollip and IRAK-M, the LPS receptor TLR4 and for the control housekeeping gene, GAPDH. Primer sequences were designed using Primer Express Software (Appiled Biosystems, UK) for amplicon product size between 100–150 base pairs (bp).

*Target*	Forward primer 5’	Size (bp)	Reverse primer 3’	Size (bp)	Product (bp)
***GAPDH***	CTGCTCCTCCTGTTCGACAGT	21	CCGTTGACTCCGACCTTCAC	23	100
***TNFα***	ACATCCAACCTTCCCAAACG	20	GCCCCCAATTCTCTTTTTGAG	22	151
***IL-10***	AGGAGGTGATGCCCCAAGCTGA	22	TCGATGACAGCGCCGTAGCCT	21	110
***IL-6***	TGGCTGCAGGACATGACAAC	20	TGAGGTGCCCATGCTACATTT	20	100
***TLR4***	AGCCCTTCACCCCGATTC	18	TAGAAATTCAGCTCCATGCATTG	23	100
***Tollip***	TCTCATGCCGTTCTGGAAAAT	21	TCACATCACAAAATGCCATGAA	22	110
***IRAK-M***	TTCAACCATGCTCGGTCATCT	21	CATACCAGGAGAACTACAGCAGA	23	137

### Western Blotting of TLR4, IRAK-M and Tollip

Cells were harvested on ice using ice-cold lysis buffer, supplemented with a protease inhibitor cocktail (1/20) and phosphatase inhibitor cocktail (1/100) (Thermo Scientific, Cramlington, UK). Protein extracts were then separated by electrophoresis in a Criterion Xt precast gel, 4–12% Bis-Tris (BIO-RAD Laboratories Ltd, Hemel Hempstead,UK), and transferred to a PVDF membrane (Thermo Scientific, Cramlington, UK). The membrane was blocked with PBS containing 0.1% v/v Tween-20 (Sigma-Aldrich, Poole, UK) and 5% w/v non-fat milk, Marvel original dried skimmed milk powder (Sainsburys, UK), and incubated with antibodies against human TLR4 (R&D Systems, Abingdon, UK), IRAK-M (Fisher Scientific, Loughborough, UK), Tollip (New England Biolabs, Herts, UK) and GAPDH (AbCam, Cambridge, UK) overnight at 4°C. After incubation with appropriate horseradish peroxidase-conjugated secondary antibodies (Bio-Rad Laboratories Ltd, Hemel Hempstead, UK) for 2 hours, immunoreactivity to blotted proteins were visualized using enhanced chemiluminescence (ECL) (GE Healthcare Life Sciences, Buckinghamshire, UK). Band density was measured using ImageJ software (available on-line, developed by NIH, USA).

### Statistical analysis

Statistical significance was analysed using a balanced analysis of variance (General Linear Model, Minitab version16 & 17) followed by a multiple comparison test. Significance was set at P values; (*p< 0.05, **p< 0.01 and ***p< 0.001 or indicated as ns = non-significant difference).

## Results

### *E*.*coli* K12-LPS induces distinct cytokine patterns in M1 and M2 macrophage subsets

**Fig 1:**
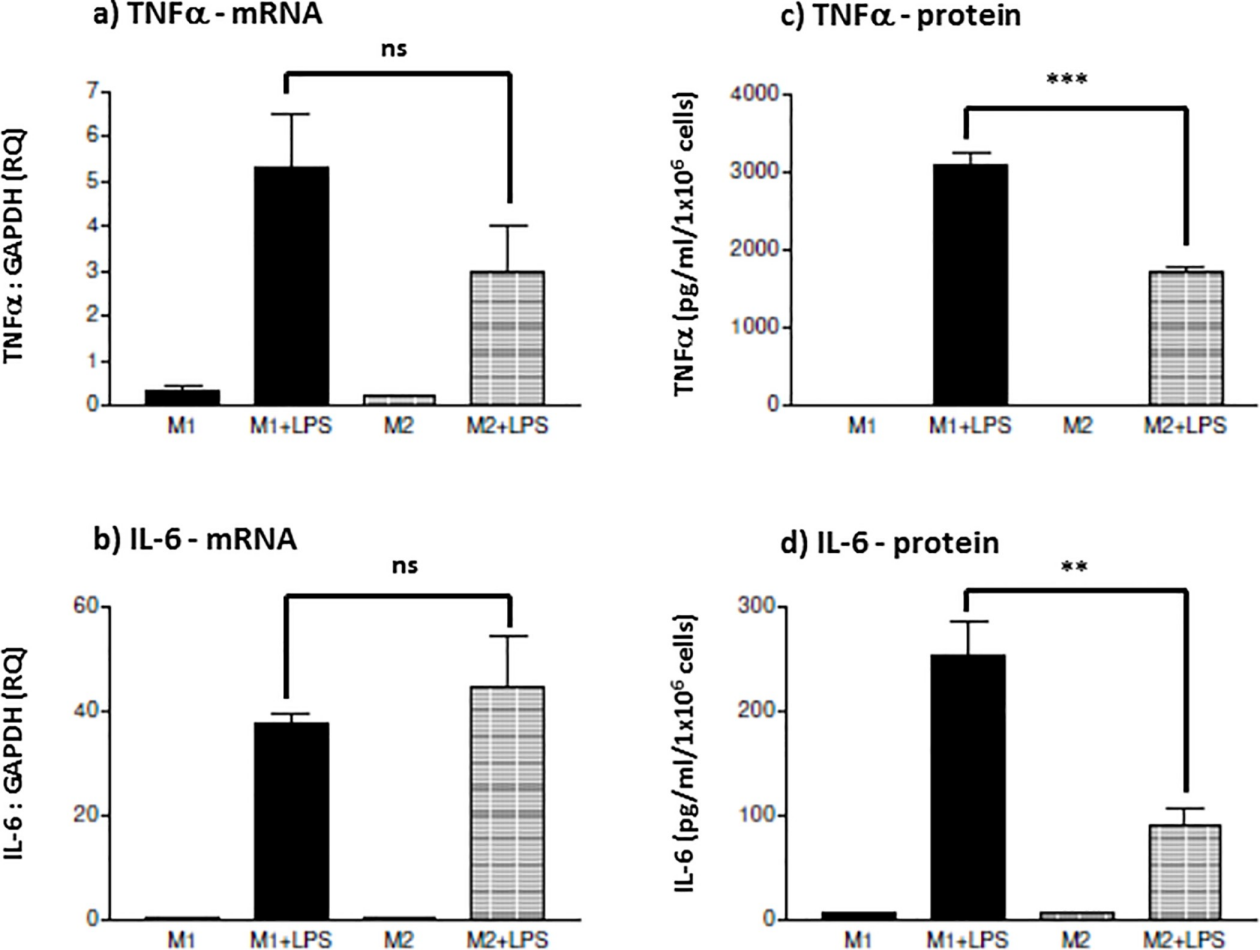
*E*.*coli* K12 LPS induces a similar pro-inflammatory cytokine profile in M1 and M2 Mφ subsets. THP-1-derived M1 and M2 M**φ**s were generated by differentiating THP-1 monocytic cells with either 25 ng/ml phorbol 12-myristate 13-acetate (PMA) for 3 days or 10 nM 1,25-(OH)_2_ vitamin D_3_ for 7 days, respectively. M1 (bold) and M2 (shaded) M**φ** subsets were stimulated with or without 100 ng/ml K12-LPS. Gene expression, mRNA, was tested in both M**φ** subsets for the expression of TNFα mRNA (a) and IL-6 mRNA (b) where mRNA level is expressed as fold change (RQ) using GAPDH as reference gene and resting cells as a calibrator sample, as described in [16] using 2^-ΔΔct^ method. Cytokine production was measured by sandwich ELISA and presented as the mean ± SD in pg/ml for TNFα (c) and IL-6 (d). Data displayed is representative of triplicate samples for n = 3 replicate experiments. Significant differences in cytokine expression and secretion between activated M1 and M2 M**φ**s are indicated as **p < 0.01, *** P < 0.001 and ns = not significant.

**Fig 2:**
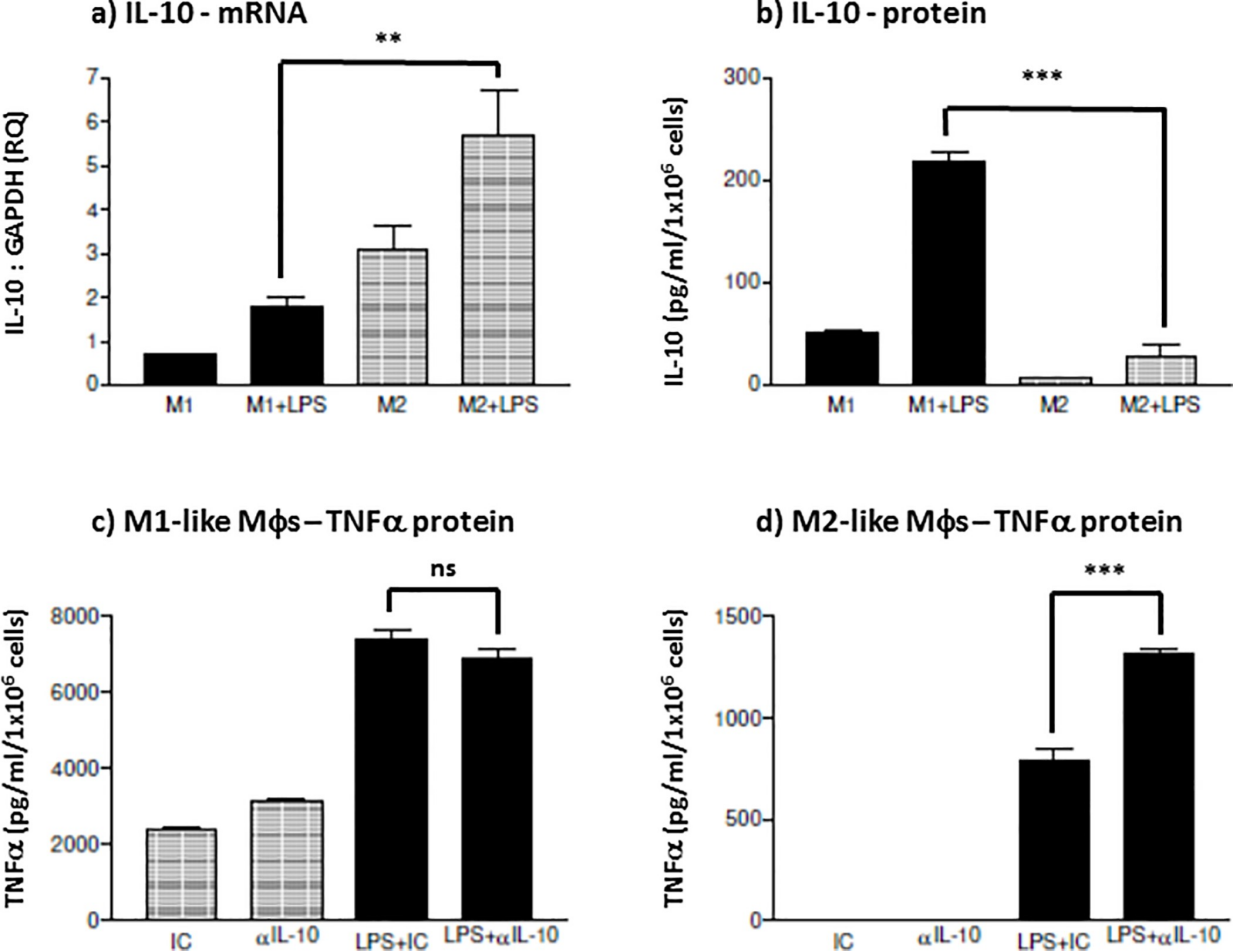
*E*.*coli* K12 LPS induces Mφ subset-specific secreted IL-10 and endogenous IL-10 activity. THP-1-derived M1 and M2 M**φ**s were generated by differentiating THP-1 monocytic cells with either 25 ng/ml phorbol 12-myristate 13-acetate (PMA) for 3 days or 10 nM 1,25-(OH)_2_ vitamin D_3_ for 7 days, respectively. M1 (bold) and M2 (shaded) M**φ** subsets were stimulated with or without 100 ng/ml K12-LPS. IL-10 gene expression of mRNA (a) is presented as fold change (RQ) using GAPDH as reference gene and resting cells as a calibrator sample, as described in [16] using 2^-ΔΔct^ method. Secretion of IL-10 (b) was measured by sandwich ELISA and presented as the mean ± SD in pg/ml. Endogenous cell-associated IL-10 activity was measured based upon the anti-inflammatory activity of IL-10 to suppress LPS-induced TNFα. M**φ**s were pre-treated with 10 μg/ml 9D7 neutralising anti-IL-10 antibody and compared to an irrelevant isotype-matched control antibody (IC) and is represented for K12-LPS-stimulated and unstimulated M1 (c) and M2 (d) macrophage TNFα secretion. TNFα is expressed as the mean± SD in pg/ml. Data displayed is representative of triplicate samples for n = 3 replicate experiments. Significant differences in cytokine expression and secretion between LPS-activated M1 and M2 M**φ**s and unstimulated controls and between isotype control and neutralising IL-10 antibody treatment are indicated as **p < 0.01, ***P < 0.001 and ns = not significant.

Mφ subsets differentially respond to LPS challenge; where M1 Mφs predominantly express pro-inflammatory cytokines and M2 Mφs predominantly express anti-inflammatory cytokines. This cytokine pattern however, can vary with different PAMP stimulation. This investigation was undertaken to characterise whether M1 and M2 Mφs reacted similarly when challenged by the enteropathogenic *E*. *coli* K12-LPS. THP-1-derived macrophages exhibited distinct cytokine patterns in M1 and M2 Mφ subsets in response to K12-LPS stimulation. Overall, when stimulated with 0.1 μg/ml K12-LPS for 18 hours, when compared to M2-like Mφs, M1-like Mφs expressed higher levels of both *de novo* mRNA (p = 0.12) and secreted TNFα protein (p = 0.0008) (Fig 1A and 1C). Although, pro-inflammatory M1-like Mφs exhibited a lower level of IL-6 mRNA compared to M2 Mφs (p = 0.09), they produced a significantly higher level of secreted IL-6 than the anti-inflammatory M2-like Mφs (p = 0.001)(Fig 1B and 1D). Considering M2 Mφs are generally described as anti-inflammatory, K12-LPS stimulation of IL-10 was investigated. M2-like Mφs expressed a higher IL-10 gene expression level than M1-like Mφs at both basal levels (unstimulated) and stimulated (p = 0.001), where LPS-induced IL-10 mRNA by M1-like Mφs was even lower than unstimulated M2-like Mφs (Fig 2A). Interestingly, K12-LPS induced minimal secretion of IL-10 by M2-like anti-inflammatory Mφs, when compared to M1-like Mφs (p = 0.0003)(Fig 2B). This apparent discrepancy in IL-10 induction and secretion between M1 and M2 Mφs is partially explained by an endogenous IL-10 activity, indeed membrane bound IL-10 has previously been described [20,21]. This endogenous activity was examined by suppressing IL-10 activity, using a neutralising anti-IL-10 antibody, and its potential ability to negate anti-inflammatory effects on TNFα production. Unstimulated Mφ subsets showed no endogenous activity (augmentation of TNFα secretion, upon neutralising IL-10 activity, Fig 2C and 2D). Upon K12-LPS stimulation however, Mφ subsets exhibited a differential expression of endogenous IL-10 activity. *E*. *coli* LPS failed to induce an endogenous suppressive IL-10 activity in M1s; TNFα induction did not display a significant change, between isotype-matched control and neutralising anti-IL-10 antibody (p = 0.176). Conversely, *E*. *coli* LPS induced an distinct endogenous IL-10 activity in M2-like Mφs. Neutralisation of IL- 10 activity increased TNFα secretion by 66%, p = 0.0002 (Fig 2C and 2D). Overall, the Mφ subsets exhibited distinct secretory cytokine patterns upon K12-LPS stimulation; M1 Mφs, induced a TNFα: IL-6: IL-10 cytokine ratio of 14:1:1, whereas K12-LPS induced a corresponding ratio of 63:3:1 by M2 Mφs (cytokine secretion between these two Mφ subsets was significant to p = 0.001 for TNFα, p = 0.001 for IL-6 and p = 0.001 for IL-10 (Figs 1 and 2). When considering the anti-inflammatory function of endogenous, cell-associated IL-10, K12-LPS induced a TNFα: IL-6: endog. IL10 ratio of 3096:254:1 and 3:0.17:1, for M1 and M2 Mφs, respectively.

### *E*. *coli* K12-LPS differentially tolerises macrophage subset cytokines

It has previously been established that M**φ** subsets exhibit differential cytokine sensitivities to ET. Tolerisation induced by the LPS of an oral pathogen, *Porphyromonas gingivalis*, resulted in suppression of M2 M**φ** production of TNFα, IL-6 and IL-10 whereas M1 Mφ production was refractory to suppression of TNFα and IL-6 (Refer to Table 2 and [5]). With regards to tolerance induction in gut mucosal macrophages and the potential to control M**φ**-driven immune fate in the GIT: inflammation/activation Vs. anti-inflammatory/regulatory, it was important to investigate M**φ** subset-specific sensitivity to tolerance induced by LPS from an enteropathogen, *Escherichia coli* strain K12. Macrophage pre-treatment with *E*. *coli* K12-LPS differentially suppressed cytokine gene expression and subsequent cytokine protein production/secretion upon re-stimulation with LPS. This homo-tolerisation of cytokine patterns is M**φ** subset-dependent. Consistent with the PG-LPS investigation, the tolerisation protocol adopted in this study utilised a 24 hour pre-treatment with K12-LPS prior to stimulation with K12-LPS for a further 18 hours. This tolerisation protocol allowed investigation of differential control of cytokine induction (determined as optimal time course for production of all cytokines TNFα, IL-6 and IL-10).

**Table 2:** *P*.*gingivalis-LPS* differentially suppresses M1 & M2 Mφ cytokines. Endotoxin tolerisation effects on PG-LPS-induced suppression of TNFα, IL-6 and IL-10 secretion by THP-1-derived M1 and M2 M**φ** subsets, where data is presented as % reduction of, or % increase over PG-LPS stimulus controls. Cytokine reduction is indicated by a downwards pointing arrow, whereas augmentation of LPS-induced cytokines are indicated by an upwards pointing arrow. Data presented is representative of n = 3 replicate experiments, already published in [5] and serves as a comparator with *E*. *coli* K12-LPS induced ET presented in Fig 3.

	(PG-LPS (Pre-treatment	/ /	PG-LPS) Stimulation)
**Cytokine:**	**TNF**α	**IL-6**	**IL-10**
M1-like Mφs	**↑ 41%**	**↓ 5%**	**↓76%**
M2-like Mφs	**↓ 98%**	**↑ 423%**	**↓ 67%**

**Fig 3:**
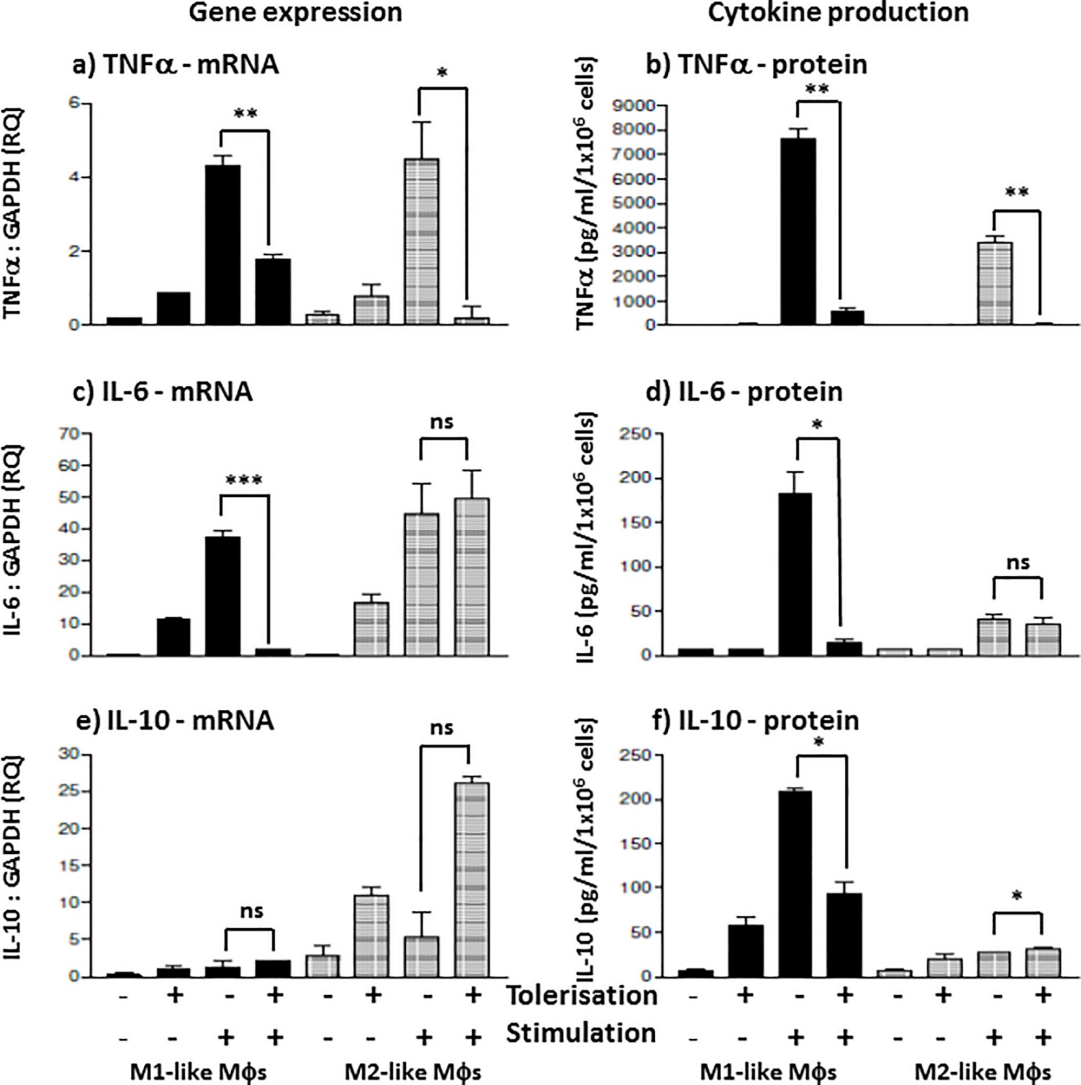
*E*.*coli* K12-LPS differentially suppresses Mφ subset cytokines secretion and gene expression. M1 (bold) and M2 (shaded) M**φ** subsets were pre-stimulated with 100 ng/ml K12-LPS for 24 hours prior to stimulation with 100 ng/ml K12-LPS incubated for a further 18 hours, indicated using (-) = no LPS, whereas (+) = LPS added for both pre-stimulated (tolerisation) and stimulated cells (stimulation). Gene expression, mRNA, was tested in both M**φ** subsets for the expression of TNFα mRNA (a), IL-6 mRNA (c) and IL-10 mRNA (e), where the mRNA level is expressed as fold change (RQ) using GAPDH as reference gene and resting cells as a calibrator sample, as described in [16] using 2^-ΔΔct^ method. Data displayed for gene expression is a representative experiment with duplicate samples for n = 3 replicate experiments. Cytokine production was measured by sandwich ELISA and presented as the mean secretion ± SD in pg/ml for TNFα (b), IL-6 (d) and IL-10 (f). Data displayed is representative of triplicate samples for n = 3 replicate experiments. Significant effects on suppression compared to the untolerised LPS stimulation control for the specified Mφ subset are indicated as * p < 0.05, ** p < 0.01, ***P < 0.001 and ns = not significant.

Gene expression of TNFα relative to GAPDH was significantly suppressed in both M1- and M2-like M**φ**s (Fig 3A). In a similar tolerisation protocol, where M**φ**s were pre-treated with K12-LPS for just 4 hours (refer to S1 Fig), a similar result was observed compared to that of the 24 hour pre-treatment; TNFα gene expression levels in M1 M**φ**s were downregulated by 60% (p = 0.045) and by 90%, (p = 0.15, ns) in M2s, respectively (S1a Fig). When pre-treated for 24 hours, TNFα mRNA levels in M1 and M2 M**φ**s were suppressed by 58% (p = 0.038) and 95% (p = 0.012), respectively (Fig 3A). K12-LPS tolerisation significantly suppressed TNFα protein production/secretion by both M1- and M2-like M**φ**s. When pre-treated for 4 hours, secreted TNFα protein was suppressed by 61%, p = 0.003 and 97%, p = 0.003 (S1B Fig); whereas upon 24 hour pre-treatment, M1 and M2 M**φ** TNFα levels were suppressed by 92% (p = 0.002) and 98% (p = 0.008), respectively (Fig 3B). There was no significant change in IL-6 gene expression compared to LPS stimulus controls (stimulation by K12-LPS without prior treatment) in M1 (p = 0.959) and M2 M**φ**s (p = 0.293) upon 4 hours pre-treatment (S1C Fig). After 24 hours pre-treatment however, M1-like M**φ**s clearly suppressed IL-6 gene expression (reduced by 95%, p = 0.002) whereas M2-like M**φ**s showed no-significant change in IL-6 gene expression (p = 0.716) (Fig 3C). Similarly, IL-6 cytokine protein production showed no significant difference in both M1 (p = 0.221) and M2 M**φ**s (p = 0.194) upon 4 hours pre-treatment (S1D Fig). Nevertheless, upon 24 hours pre-treatment, K12-LPS clearly suppressed M1-like M**φ** induction of IL-6 (reduced by 92%, p = 0.021) and showed no significant suppression in IL-6 production (reduced by 14%, p = 0.364) by M2-like M**φ**s (Fig 3D).

Interestingly, gene expression of the anti-inflammatory cytokine, IL-10, showed significant upregulation by both M**φ** subsets upon 4 hour pre-treatment tolerisation. The increase level in IL-10 gene expression was 110% higher in both M1- and M2-like M**φ**s, p = 0.006 and p = 0.005, respectively (S1E Fig). IL-10 gene expression did not show any significant change upon tolerisation in both M**φ** subsets after 24 hours of pre-treatment (p = 0.42), however both subsets exhibited an augmentation trend in LPS-induced IL-10 mRNA (Fig 3E). Pro-inflammatory M1-like M**φ**s showed no change (p = 0.785) in IL-10 protein production when they were pre-treated by K12-LPS for 4 hours; anti-inflammatory M2-like Mφs however, displayed an up-regulation of IL-10 by 176% (p = 0.035) (S1F Fig). Secretion of IL-10 by pro-inflammatory M1 M**φ**s was clearly suppressed (56%, p = 0.002) when pre-treated with K12-LPS for 24 hours compared to positive controls. On the other hand, anti-inflammatory M2 M**φ**s displayed a slight but significant increase in IL-10 cytokine secretion (p = 0.039) upon tolerisation over stimulus controls (Fig 3F).

### *E*. *coli* K12-LPS tolerisation of Mφ subsets is independent of a down-regulation in TLR4 protein

**Fig 4:**
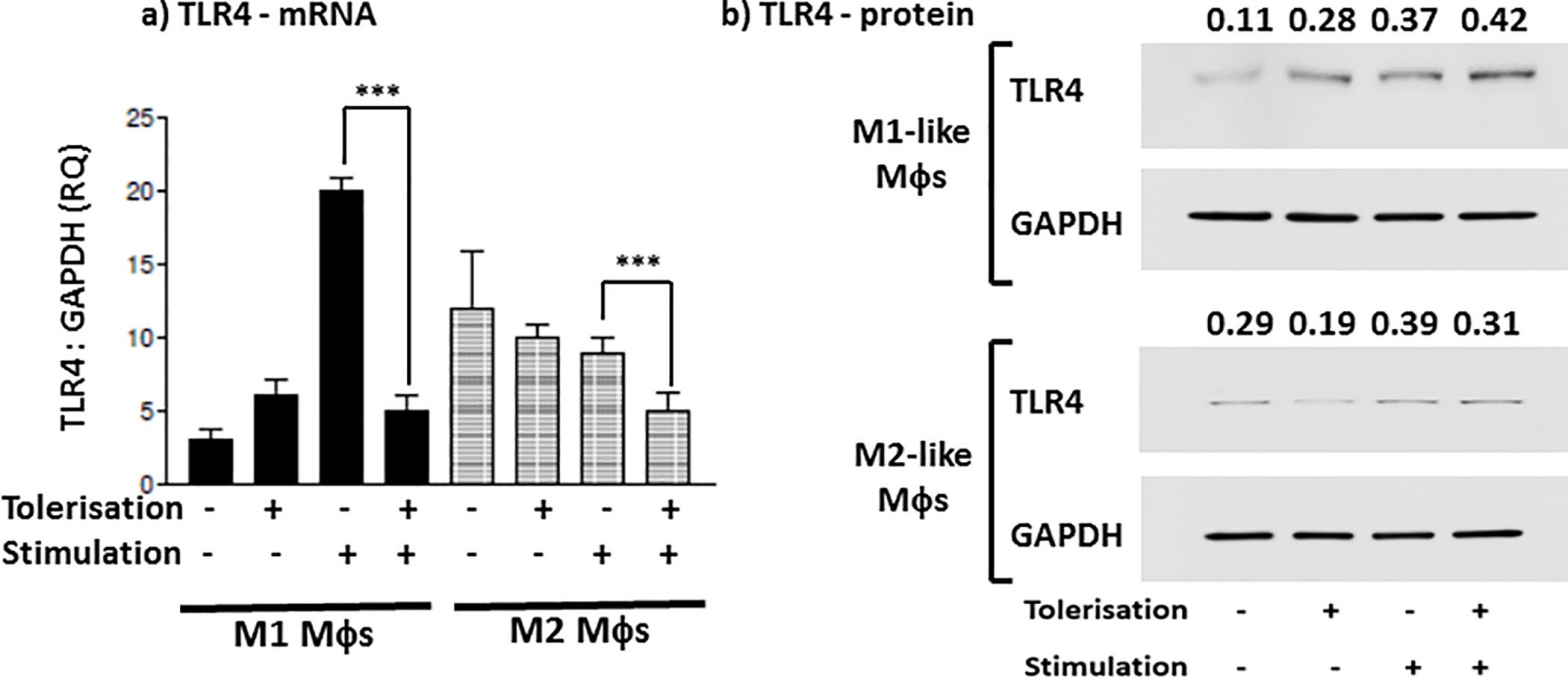
*E*.*coli* K12-LPS tolerisation of Mφ subsets is independent of a down-regulation in TLR4 protein. M1 (bold) and M2 (shaded) M**φ** subsets were pre-stimulated with 100 ng/ml K12-LPS for 24 hours prior to stimulation with 100 ng/ml K12-LPS incubated for a further 18 hours, indicated using (-) = no LPS, whereas (+) = LPS added for both pre-stimulated (tolerisation) and stimulated cells (stimulation). Gene expression, mRNA, was tested in both M**φ** subsets for the expression of TLR4 mRNA (a), where the mRNA level is expressed as fold change (RQ) using GAPDH as reference gene and resting cells as a calibrator sample, as described in [16] using 2^-ΔΔct^ method. Data displayed for gene expression is a representative experiment with duplicate samples for n = 3 replicate experiments. Significant effects on suppression (+/+) compared to the untolerised LPS stimulation control (-/+) for the specified M**φ** subset are indicated as ***P < 0.001. TLR4 protein (b) was detected by western blotting and levels of GAPDH served as internal controls. The relative band density ratio of TLR4 protein compared to the GAPDH house-keeping protein loading control is indicated numerically above the appropriate sample detected on the blot. Data displayed is a representative blot of n = 3 independent replicate experiments.

Down-regulation in TLR4 surface expression is recognised as a mechanistic response to ET. To establish an understanding of ET mechanisms utilised in M1 and M2 M**φ** subsets, it was important to investigate the influence of ET on TLR4, the pattern recognition receptor to the K12-LPS agonist. Upregulation of TLR4 mRNA was observed in M1 M**φ**s stimulated with K12-LPS. After pre-stimulation, challenge with the same LPS for an additional 24 hours, markedly decreased the levels of TLR4 gene expression in M1-like M**φ**s by 75% compared with LPS-stimulated cells (p = 0.001). Additionally, M2-like M**φ**s also showed a significant downregulation of TLR4 gene expression by 50% upon LPS pre-stimulation/stimulation (p = 0.001) (Fig 4A). No significant changes in TLR4 gene expression were observed in M1 (p = 0.1) and M2s (p = 0.5) for the 4 hour pre-treatment (S2A Fig). Interestingly however, was the fact that these ET effects on TLR4 mRNA were not paralleled by TLR4 protein. TLR4 protein was upregulated in M1-like M**φ**s upon stimulation with the same pre-treatment challenge compared to the stimulus control. The band density of positive stimulus control, compared to the GAPDH house-keeping protein loading control, increased by 14% from 0.37 to 0.42, upon tolerisation. On the other hand, in tolerised M2-like M**φ**s, TLR4 was down-regulated by 20% upon stimulation with the same pre-treatment/treatment challenges, from stimulus control levels of 0.39 to 0.31 (Fig 4B).

### Macrophage subsets exhibit differential regulation of IRAK-M and Tollip upon endotoxin tolerisation

**Fig 5:**
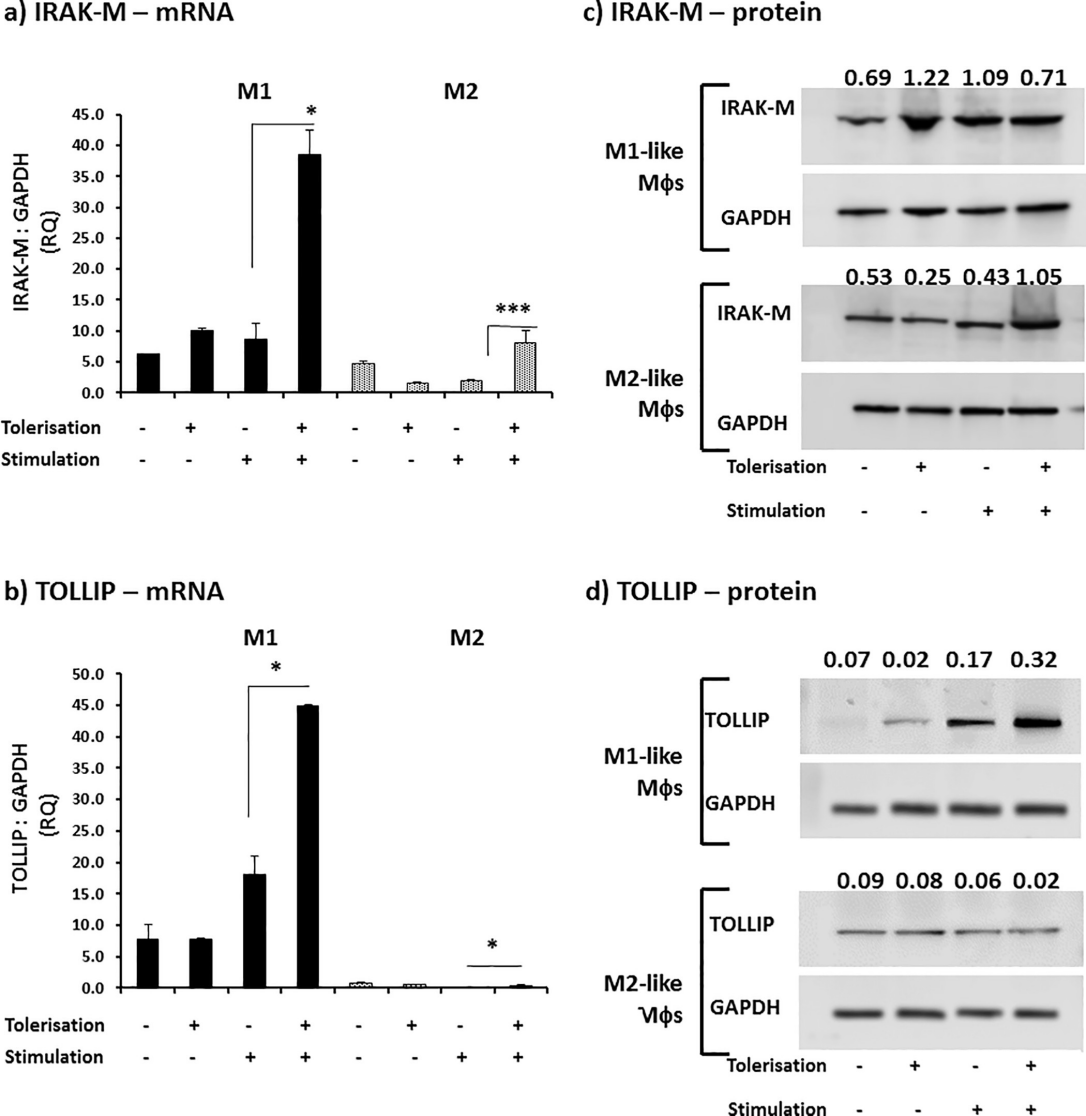
Endotoxin-tolerisation of Mφ subsets differentially regulates IRAK-M and Tollip. M1 (bold) and M2 (shaded) M**φ** subsets were pre-stimulated with 100 ng/ml K12-LPS for 24 hours prior to stimulation with 100 ng/ml K12-LPS incubated for a further 18 hours, indicated using (-) = no LPS, whereas (+) = LPS added for both pre-stimulated (tolerisation) and stimulated cells (stimulation). Gene expression, mRNA, was tested in both M**φ** subsets for the expression of IRAK-M mRNA (a) and Tollip mRNA (b), where the mRNA level is expressed as fold change (RQ) using GAPDH as reference gene and resting cells as a calibrator sample, as described in [16) using 2^-ΔΔct^ method. Data displayed for gene expression is a representative experiment with duplicate samples of n = 3 replicate experiments. Significant effects on suppression (+/+) compared to the untolerised LPS stimulation control (-/+) for the specified Mφ subset are indicated as * P<0.05, *** P<0.001. IRAK**-**M (c) and Tollip (d) protein was detected by western blotting and levels of GAPDH served as internal controls. The relative band density ratio of IRAK-M and Tollip protein compared to the GAPDH house-keeping protein loading control is indicated numerically above the appropriate sample detected on the blot. Data displayed is a representative blot of n = 3 independent replicate experiments.

Previous results presented in this investigation indicate a differential sensitivity of M**φ** subset induced cytokines to ET, and that this differential cytokine suppression was independent of a down-regulation in the LPS receptor, TLR4. As a consequence of this lack of suppression of TLR4, mechanistic focus was changed to intracellular negative regulators of TLR4 signalling, such as IRAK-M and Tollip. Indeed, K12-LPS ET selectively up-regulated the TLR negative regulators IRAK-M and Tollip in a subset-specific manner. Upon tolerance induction, M**φ** subsets exhibited a clear up-regulation in the gene expression of the negative regulators, IRAK-M and Tollip mRNA, with the greatest increase observed in M1 M**φ**s. In the case of the 4 hour pre-treatment tolerance induction, M1-like M**φ**s showed a significant up-regulation for IRAK-M mRNA 160% (p = 0.04), whereas M2-like M**φ**s showed no difference in IRAK-M mRNA expression levels (p = 0.25, S2B Fig). For 24 hour pre-treatment, both endotoxin-tolerised M1- and M2-like M**φ**s showed a significant up-regulation for IRAK-M mRNA by 330% (p = 0.024) and 300% (p = 0.001), respectively (Fig 5A). The differential M**φ** subset-specific response was observed at the IRAK-M protein level. Interestingly, ET-induced M1-like M**φ**s failed to present any appreciable change in IRAK-M protein level, whereas M2-like M**φ**s showed a clear upregulation in IRAK-M protein. This was reinforced by protein band densitometry expressed as the relative IRAK-M band density to the loading control density of GAPDH in the same sample; where the relative band density for the LPS control exhibited by M1 M**φ**s was 1.089 which decreased by 35% to 0.712 upon tolerisation. In the case of M2 M**φ**s, the relative density increased from 0.431 to 1.053, thus IRAK-M protein was increased by 144% by tolerisation pre-stimulation over control LPS stimulation (Fig 5C). Thus, tolerisation induced a matched up-regulation in IRAK-M mRNA and protein in M2 M**φ**s, whereas ET induced up-regulation in IRAK-M mRNA in M1 M**φ**s was not matched by that of IRAK-M protein. Interestingly, the opposite case was observed with Tollip results. M1-like M**φ**s displayed a significant up-regulation by 140% (p = 0.02) and M2 M**φ**s presented a down-regulation by 60% (p = 0.009) of Tollip mRNA after 4 hours pre-treatment tolerisation (S2C Fig). Although endotoxin-tolerised M1- and M2-like M**φ**s clearly up-regulated Tollip mRNA expression after 24 hours pre-treatment tolerisation by 140% (p = 0.011) and 300% (p = 0.025), respectively (Fig 5B), M1-like M**φ**s only, exhibited an appreciable augmentation in the protein level of Tollip. The relative band density for Tollip protein for the LPS stimulus control exhibited by M1 M**φ**s was 0.167 which increased to 0.322 upon tolerisation, whereas for M2 M**φ**s, the relative density decreased from 0.061 to 0.014, thus Tollip protein was increased by 93% by tolerisation pre-stimulation over control LPS stimulation levels in M1 pro-inflammatory M**φ**s and decreased by 77% in M2s, although this M2 result is not clearly visible in blot (Fig 5D).

## Discussion

M**φ** subsets have previously been shown to exhibit a differential sensitivity to ET induced in response to PG-LPS, derived from the oral keystone pathogen associated with chronic periodontitis, *Porphyromonas gingivalis* [5]. In brief, CD14^lo^ M2 M**φ**s, which resemble homeostatic, tissue-resident mucosal M**φ**s, were sensitive to PG-LPS-induced ET (suppression of TNFα, IL-1β, IL-6, IL-10 and NFκB) whereas CD14^hi^ M1 Mφs, representative of pro-inflammatory mucosal Mφs, were generally refractory to tolerance induction by PG-LPS (little, if any suppression of TNFα, IL-1β, IL-6 and NFκB). This investigation aimed to investigate whether the same, or a different, response to ET could be observed between the M**φ** subsets when tolerised by the LPS from a known gut mucosal enteropathogen, *E*. *coli* K12.

Firstly, *E*.*coli* K12 LPS-induced pro-inflammatory and anti-inflammatory cytokine production is dependent on the route of M**φ** differentiation. Both subsets exhibited *de novo* expression and secretion of pro-inflammatory cytokines; where M1-like M**φ**s were characterised as TNFα^hi^ and IL-6^hi^ and M2-like Mφs were TNFα^med^ and IL-6^lo^. At first glance, the induction of the secreted anti-inflammatory cytokine, IL-10, did not conform to the perception that M1 M**φ**s are generally pro-inflammatory whereas M2 M**φ**s are anti-inflammatory; M1-like M**φ**s secreted higher levels of IL-10 protein than M2-like M**φ**s, whereas M2-like M**φ**s did exhibit a significantly higher level of both IL-10 mRNA and endogenous cell-associated IL-10 activity. Secondly, in contrast to PG-LPS tolerisation, ET induced by *E*. *coli* K12-LPS failed to demonstrate a differential subset-specific response, whereas displayed differential sensitivity to tolerance induction of inflammatory cytokines. Both K12-LPS tolerised M1- and M2-like M**φ** subsets exhibited suppression of the pro-inflammatory cytokine (TNFα: both mRNA and protein secretion), however a differential subset-specific response was observed for IL-6 and IL-10. M1 M**φ**s were sensitive to suppression of IL-6 and IL-10 and in contrast, M2 M**φ**s were refractory to suppression of IL-6 and IL-10. This trend for cytokine protein secretion paralleled IL-6 mRNA, however IL-10 mRNA did not follow that of IL-10 secreted protein; where IL-10 mRNA was non-significantly up-regulated in M2 M**φ**s, yet relatively unchanged in M1 M**φ**s. Thirdly, suppression of these inflammatory cytokines appeared to be independent of a down-regulation of TLR4 protein in both M**φ** subsets, however both subsets exhibited a significant decrease in TLR4 mRNA. Finally, and arguably most importantly, a differential subset-specific regulation was observed for endogenous negative regulators of TLR4-mediated signalling. There was a differential up-regulation in IRAK-M and Tollip protein upon K12-LPS ET induction; Tollip was upregulated in M1 M**φ**s and IRAK-M was upregulated in M2s.

In this study, M1 (pro-inflammatory) and M2 (anti-inflammatory) M**φ** subsets were modelled using PMA- and Vitamin D_3_-differentiation of the human THP-1 cell line. Upon stimulation by *E*.*coli* K12-LPS, these M**φ** subsets exhibited similar cytokine profiles to those reported for primary peripheral blood-derived M**φ**s. TNFα cytokine production was higher in M1s than M2s in response to stimulation by the TLR4 agonist, K12-LPS (Fig 1A and 1C). Although it is believed that IL-6 is pro-inflammatory, it has also been described to display anti-inflammatory properties. The nature of IL-6 production and function is probably being reflected in different signalling profiles [22]. Anti-inflammatory effects of IL-6 is generally reflected by initiation of SOCS proteins and STAT-3 activation [23] and reviewed in [24]. In fact, the STAT-3-inducible molecule, SOCS-3 is associated with M1 classical M**φ** polarisation and can suppress the anti-inflammatory signal and expression of IL-6 and IL-10. On the other hand, SOCS-3 knockdown favours M2 polarisation [25]. Additionally, IL-6 also induces IL-1Ra, soluble p55 TNF-R and suppresses NFκB; all of which are suggestive of IL-6 promoting an M2 Mφ phenotype [22,26]. Therefore, the mutual association between SOCS-3 and STAT-3 would seem to control pro- or anti-inflammatory outcome of IL-6, its production, and the polarisation of Mφs between M1 and M2 subsets. M1 Mφs produced a significantly high level of IL10, although it was expected that the higher level of IL-10 production would be by M2 Mφs. However, it has been observed in previous studies, and in our laboratory, that IL-10 in M2 Mφs is expressed endogenously or as membrane bound protein [13,20].

It is well established that multiple stimulation by LPS can induce ET. The use of the pre-stimulation/stimulation protocol by K12-LPS is employed to investigate TLR4-mediated homo-tolerisation. Pro-inflammatory and anti-inflammatory Mφs exhibited a comparable level of TNFα suppression upon 24 hour priming pre-stimulation, followed by stimulation. The TNFα suppression in protein secretion was supported by a similar level of suppression shown in TNFα mRNA. Additionally, the suppression of IL-6 production in M1 Mφs might be associated with TNFα, in that IL-6 mRNA and protein suppression followed the profile of TNFα after the 24 hour pre-treatment protocol whereas 4 hour pre-treatment failed to exhibit significant suppression in M1 Mφs (S1 Fig). This delayed suppression of IL-6 behind the TNFα response may be indicative of IL-6 production being TNFα-dependent in M1 Mφs. With regards IL-10 mRNA, M2 Mφs exhibited the higher level of expression compared to M1s, whereas tolerisation induced a differential response between these Mφ subsets: tolerisation induced an increase in IL-10 mRNA in M2s whereas only a small increase for M1s. This trend was not reproduced however, when reviewing IL-10 protein secretion, where IL-10 secretion was selectively suppressed in M1s. This differential response between M1 and M2 Mφs, may be indicative of both differences in mRNA stability and endogenous, membrane-associated IL-10 activity. A potential differential stability of IL-10 mRNA and protein trafficking resulting in either secreted IL-10 or membrane-bound IL-10 activity, might go some way to explain why M1 Mφs secreted higher levels of IL-10 protein than M2 Mφs, despite the fact that M1-induced IL-10 secretion was suppressed by repeated LPS exposure and that M2 Mφs expressed the higher relative expression of IL-10 mRNA. ET is often related with over-secretion of anti-inflammatory cytokines, such as IL-10 and TGF-β, which contribute to the deactivation of Mφs and the suppression of pro-inflammatory cytokine production [1]. Thus, ET more likely represents a selective reprogramming, intended at reducing inflammatory damage [27]. This differential cytokine production or selective reprogramming, potentially converting an M1 cytokine phenotype to that of a regulatory M2 subset phenotype, is likely to be associated with distinct signaling pathways.

*E*. *coli* LPS demonstrated different abilities to modulate expression levels of members of the TLR family. These results show that, in response to repeated challenge by K12-LPS, the expression of TLR4 mRNA was significantly down-regulated by both Mφ subsets; this was not however, reflected by the protein levels, where M1-like Mφs showed a small up-regulation in TLR4 receptor whereas M2 Mφs exhibited a small decrease. This is generally contradictory to ET mechanisms described in Mφs [28], however may be indicative of a subset-specific sensitivity to tolerisation. The cytokine expression profiles secreted by LPS tolerised Mφs could be related to diversity of regulatory mechanisms associated with TLR4-mediated signaling. As a TLR4 agonist, K12-LPS, can activate both MyD88-dependent and MyD88-independent pathways and present clear suppression in cytokine production in endotoxin-tolerised Mφs, this is in stark contrast to TLR2, which signals only through a MyD88-dependent pathway [11]. Endogenous LPS-recognition utilizes an endosomal TRIF/TRAM-adaptor (Myd88-independent) pathway, which results in the activation of IRF3 and the expression of type I IFNs. It has long since been established that type I IFNs can induce IL-10 production [29,30], hence influencing a regulatory phenotype; this may go some way to explaining the differential cytokine responses to ET in distinct Mφ subsets.

In addition to the contribution of LPS-reception events (receptors and TIR-interacting adaptor molecules), that influence ET-mediated cytokine responses (hence control of inflammation) in these Mφ subsets, it was important to investigate post-reception signal regulation tolerogenic mechanisms. Therefore, further experiments compared and contrasted the modulation in gene expression and protein level of negative regulators of TLR4 signaling in endotoxin tolerised M1- and M2-like Mφs. The negative regulators, which have been studied, were IRAK-M and Tollip (Fig 5 and S2 Fig). IRAK-M is a negative regulator, preferentially expressed in monocytes and macrophages, which inhibits the dissociation of the active kinase isoforms, IRAK-1 and IRAK-4, from the TLR4-Myd88 complex, effectively suppressing signaling through NFκB and expression of pro-inflammatory such as TNFα and IL-1β [31–33]. Indeed, IRAK-1 kinase activity is reduced in LPS-tolerised human and murine Mφs [28,34], whereas knockout of IRAK-M impairs Mφ ET and results in augmentation in LPS-induced activation of NFκB and MAPK [32]. In addition to dissociation of IRAKs-1 and -4, it has also been reported that IRAK-M might inhibit signal transduction between Myd88 and IRAK-1, thus preventing complex formation with downstream reduction in cytokine expression [35]. Although IRAK-M expression has been reported in both murine and human models of ET (reviewed in [1]), no investigations have suggested a potential difference in IRAK-M-mediated ET induced in distinct Mφ subsets. Tollip is associated with both TLR2 and TLR4, playing an inhibitory role in TLR-mediated cell activation, through its capability to suppress the activity of IL-1 receptor-associated kinase (IRAK) after TLR ligation/reception [36]. Indeed, human Tollip was described to regulate TLR2 and TLR4 signaling with the consequent suppression of the pro-inflammatory cytokines TNFα and IL-6 whilst up-regulating the anti-inflammatory cytokine, IL-10 [37]. This is suggestive of Tollip exerting its regulatory effects on inflammation through both direct suppression of inflammatory cytokine expression and their down-regulation of expression and functionality by the induction of IL-10 [38]. Additionally, this Tollip-mediated tolerisation is likely to be context dependent, where high-dose LPS induces Tollip expression and facilitates resolution of inflammation [39,40], whereas extremely low-dose LPS induced a cell stress response through clearing Tollip, blocking lysosomal fusion events, hence perpetuating chronic inflammation [41]. Interestingly, there was a distinct difference in gene expression of these negative regulators between tolerised M1 Mφs and M2 Mφs, where both regulators were up-regulated by LPS tolerisation, with the highest levels observed in M1 Mφs. This augmentation of gene expression however, was not necessarily translated to protein levels of IRAK-M and Tollip: Tollip was up-regulated in ET induced in M1-like Mφs whereas IRAK-M was up-regulated in M2s. It is suggestive that this differential utilisation of negative regulators of TLR signalling may have an influence on the relative ET responses of these Mφ subsets with respect to their expression, secretion and responsiveness to TNFα or to IL-6 and IL-10. The differential involvement of IRAK-M and Tollip in Mφ subset responses to ET and their role as upstream negative regulators of NFκB-driven signalling responses [32,37] is indicative of both a differential NFκB signal dependence of these distinct subsets, but may also indicate a role for NFκB as a molecular discriminator of Mφ polarisation. Indeed, p65 NFκB subunit inhibition has been demonstrated to favour anti-inflammatory M2-like Mφ polarisation [42]; where p65 NFκB subunit favours M1 polarisation and activity and p50/p50 NFκB drives M2 polarisation [43]. Just how this differential utilization of negative regulators in distinct Mφ subsets determines inflammatory cytokine responses will be the subject of future investigations aimed at selective suppression of Mφ subsets associated with inflammatory pathology and cancer.

In contrast to previously published investigations where M1- and M2-like Mφ subsets exhibited a differential sensitivity of TNFα secretion to ET induced by PG-LPS [5], no such dichotomy in TNFα response was seen in the same Mφ subsets where ET was induced by *E*. *coli* K12-LPS. With regards IL-10 secretion however, both subsets were sensitive to ET induced by PG-LPS whereas IL-10 (and additionally, IL-6) were differentially regulated by K12-LPS-induced ET in M1- and M2-like Mφ subsets. This differential Mφ subset response between ET induced by PG-LPS and K12-LPS would appear to be independent of a down-regulation in TLR4 protein and is suggestive of subtle Mφ subset- and bacterial species LPS-dependent effects being associated with signalling downstream of TLR reception events. As a consequence, this study has described a differential, subset-dependent ET-induced up-regulation in distinct negative regulators of TLR signal transduction pathways; where Tollip was augmented in M1 Mφs and IRAK-M in M2s upon K12-LPS ET. Manipulation of the expression and binding activities of such regulators is likely to have a significant and discriminatory effect on Mφ subset cytokine phenotype and thus, manipulation of ET may exert a profound effect in the regulation of inflammation via polarisation of Mφ subsets between pro-inflammatory (M1-like) and anti-inflammatory (M2-like) phenotypes.

## Supporting information

S1 Dataset (XLSX)

S1 Fig Short-term pre-treatment with *E*.*coli* K12-LPS differentially suppresses Mφ subset cytokine secretion and gene expression. M1 (bold) and M2 (shaded) Mφ subsets were pre-stimulated with 100 ng/ml K12-LPS for 4 hours prior to stimulation with 100 ng/ml K12-LPS incubated for a further 18 hours, indicated using (-) = no LPS, whereas (+) = LPS added for both pre-stimulated (tolerisation) and stimulated cells (stimulation). Gene expression, mRNA, was tested in both Mφ subsets for the expression of TNFα mRNA (a), IL-6 mRNA (c) and IL-10 mRNA (e), where the mRNA level is expressed as fold change (RQ) using GAPDH as reference gene and resting cells as a calibrator sample, as described in [16] using 2^-ΔΔct^ method. Data displayed for gene expression is a representative experiment with duplicate samples for n = 3 replicate experiments. Cytokine production was measured by sandwich ELISA and presented as the mean secretion ± SD in pg/ml for TNFα (b), IL-6 (d) and IL-10 (f). Data displayed is representative of triplicate samples for n = 3 replicate experiments. Significant effects on suppression compared to the untolerised LPS stimulation control for the specified Mφ subset are indicated as * p < 0.05, ** p < 0.01 and ns = not significant.
(TIF)

S2 Fig Short-term pre-treatment *E*.*coli* K12-LPS tolerisation of Mφ subsets differentially regulates IRAK-M, Tollip and TLR4 gene expression. M1 (bold) and M2 (shaded) Mφ subsets were pre-stimulated with 100 ng/ml K12-LPS for 4 hours prior to stimulation with 100 ng/ml K12-LPS incubated for a further 18 hours, indicated using (-) = no LPS, whereas (+) = LPS added for both pre-stimulated (tolerisation) and stimulated cells (stimulation). Gene expression, mRNA, was tested in both Mφ subsets for the expression of TLR4 mRNA (a), IRAK-M mRNA (b) and Tollip mRNA (c), where the mRNA level is expressed as fold change (RQ) using GAPDH as reference gene and resting cells as a calibrator sample, as described in [16] using 2^-ΔΔct^ method. Data displayed for gene expression is a representative experiment with duplicate samples of n = 3 independent replicate experiments. Significant effects on suppression (+/+) compared to the untolerised LPS stimulation control (-/+) for the specified Mφ subset are indicated as* P<0.05, ** P<0.01 and ns, not significant.
(TIF)
