# Supplementary figures and images for: Macrophage subsets exhibit distinct E. coli-LPS tolerisable cytokines associated with the negative regulators, IRAK-M and Tollip
Source: PLoS One. 2019 May 23;14(5):e0214681. doi: 10.1371/journal.pone.0214681 (PMC6533032; doi:10.1371/journal.pone.0214681)

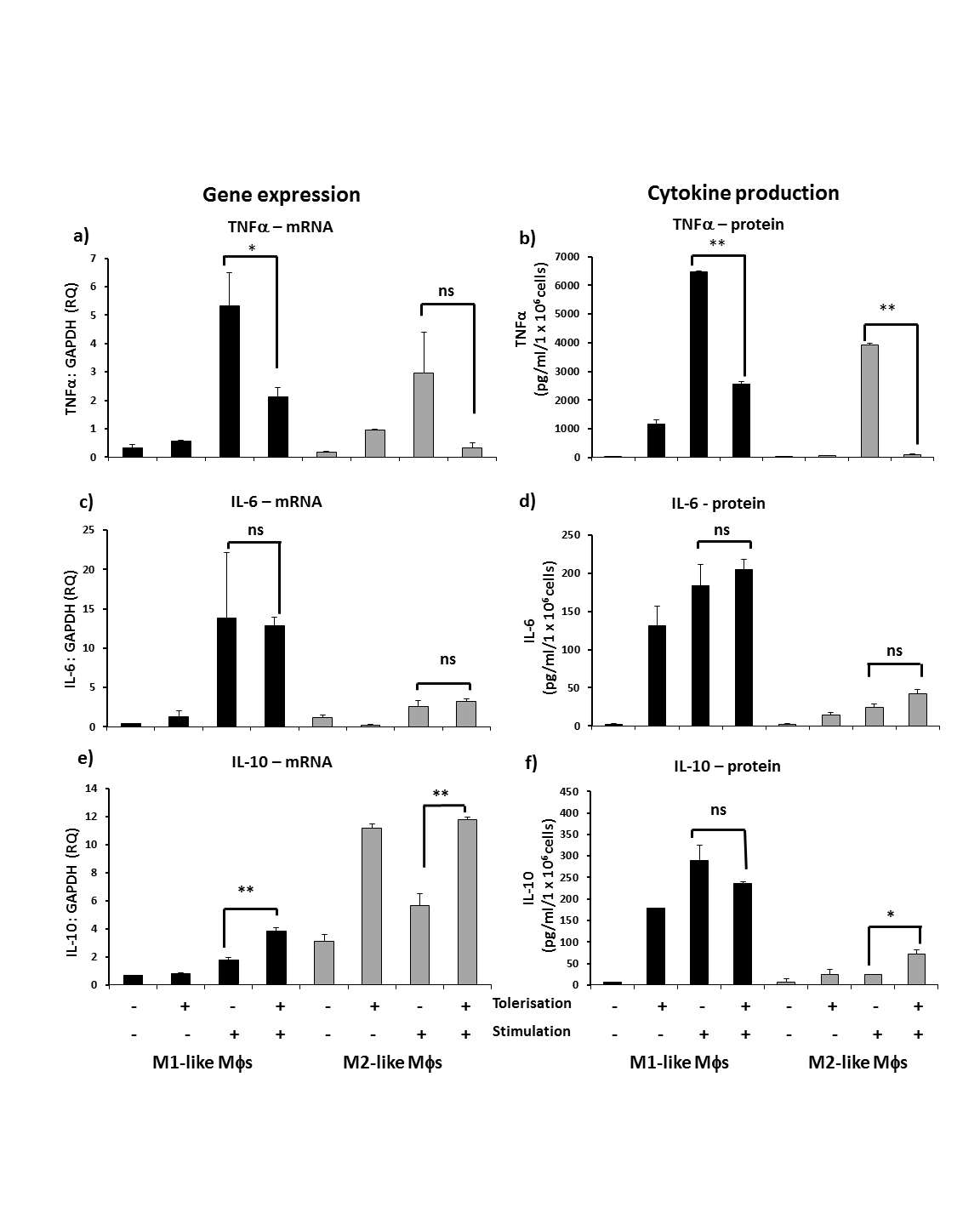

Supplement: S1 Fig — M1 (bold) and M2 (shaded) Mφ subsets were pre-stimulated with 100 ng/ml K12-LPS for 4 hours prior to stimulation with 100 ng/ml K12-LPS incubated for a further 18 hours, indicated using (-) = no LPS, whereas (+) = LPS added for both pre-stimulated (tolerisation) and stimulated cells (stimulation). Gene expression, mRNA, was tested in both Mφ subsets for the expression of TNFα mRNA (a), IL-6 mRNA (c) and IL-10 mRNA (e), where the mRNA level is expressed as fold change (RQ) using GAPDH as reference gene and resting cells as a calibrator sample, as described in [16] using 2-ΔΔct method. Data displayed for gene expression is a representative experiment with duplicate samples for n = 3 replicate experiments. Cytokine production was measured by sandwich ELISA and presented as the mean secretion ± SD in pg/ml for TNFα (b), IL-6 (d) and IL-10 (f). Data displayed is representative of triplicate samples for n = 3 replicate experiments. Significant effects on suppression compared to the untolerised LPS stimulation control for the specified Mφ subset are indicated as * p < 0.05, ** p < 0.01 and ns = not significant. (TIF) [file pone.0214681.s002.tif]

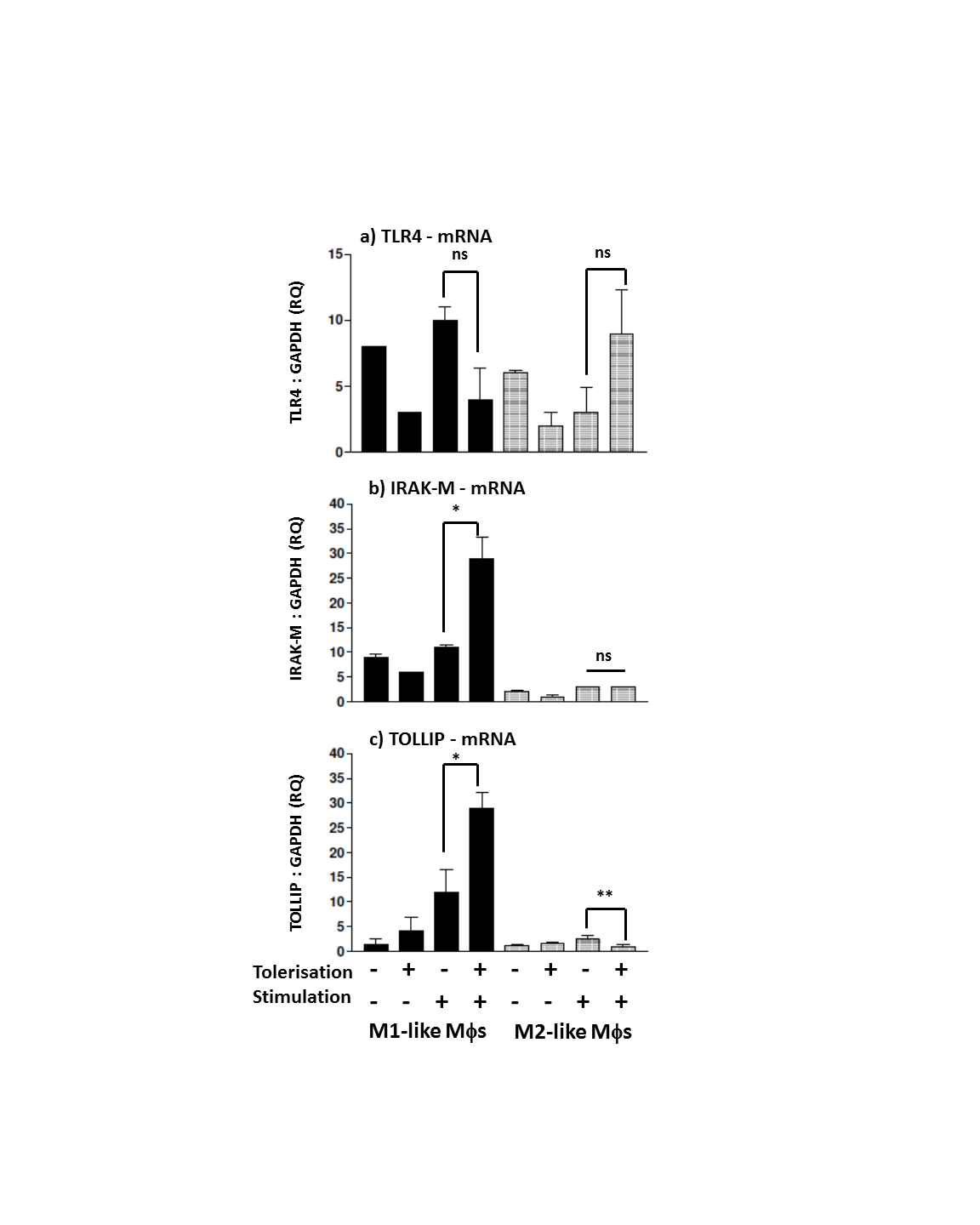

Supplement: S2 Fig — M1 (bold) and M2 (shaded) Mφ subsets were pre-stimulated with 100 ng/ml K12-LPS for 4 hours prior to stimulation with 100 ng/ml K12-LPS incubated for a further 18 hours, indicated using (-) = no LPS, whereas (+) = LPS added for both pre-stimulated (tolerisation) and stimulated cells (stimulation). Gene expression, mRNA, was tested in both Mφ subsets for the expression of TLR4 mRNA (a), IRAK-M mRNA (b) and Tollip mRNA (c), where the mRNA level is expressed as fold change (RQ) using GAPDH as reference gene and resting cells as a calibrator sample, as described in [16] using 2-ΔΔct method. Data displayed for gene expression is a representative experiment with duplicate samples of n = 3 independent replicate experiments. Significant effects on suppression (+/+) compared to the untolerised LPS stimulation control (-/+) for the specified Mφ subset are indicated as* P<0.05, ** P<0.01 and ns, not significant. (TIF) [file pone.0214681.s003.tif]
